# Current Approaches for Diagnosis of Influenza Virus Infections in Humans

**DOI:** 10.3390/v8040096

**Published:** 2016-04-12

**Authors:** Sai Vikram Vemula, Jiangqin Zhao, Jikun Liu, Xue Wang, Santanu Biswas, Indira Hewlett

**Affiliations:** Laboratory of Molecular Virology, Center for Biologics Evaluation and Research, Food and Drug Administration, Silver Spring, MD 20993, USA; vikisai@gmail.com (S.V.V.); jiangqin.zhao@fda.hhs.gov (J.Z.); Jikun.Liu@fda.hhs.gov (J.L.); Xue.Wang@fda.hhs.gov (X.W.); Santanu.Biswas@fda.hhs.gov (S.B.)

**Keywords:** Influenza diagnostics, Immunoassay, Influenza viruses, hemaglutinin, neuraminidase, subtype, next-generation sequencing (NGS)

## Abstract

Despite significant advancement in vaccine and virus research, influenza continues to be a major public health concern. Each year in the United States of America, influenza viruses are responsible for seasonal epidemics resulting in over 200,000 hospitalizations and 30,000–50,000 deaths. Accurate and early diagnosis of influenza viral infections are critical for rapid initiation of antiviral therapy to reduce influenza related morbidity and mortality both during seasonal epidemics and pandemics. Several different approaches are currently available for diagnosis of influenza infections in humans. These include viral isolation in cell culture, immunofluorescence assays, nucleic acid amplification tests, immunochromatography-based rapid diagnostic tests, *etc.* Newer diagnostic approaches are being developed to overcome the limitations associated with some of the conventional detection methods. This review discusses diagnostic approaches currently available for detection of influenza viruses in humans.

## 1. Introduction

Influenza, also known as the flu, is a respiratory illness caused by viruses belonging to the family *Orthomyxoviridae*. This family consists of four influenza virus genera (*influenza virus A*, *influenza virus B*, *influenza virus C*, and *influenza virus D*) that are classified based on differences in their internal glycoproteins nucleoprotein (NP) and matrix (M). Influenza type A viruses can infect humans, birds, pigs, horses, and other animals, while influenza B and C viruses are found only in humans. Influenza viruses contain a single stranded negative sense RNA genome that encodes 11 proteins. Based on the viral surface glycoproteins hemagglutinin (HA) and neuraminidase (NA), influenza A viruses are divided into various subtypes. There are 18 HA (H1–H18) and 11 NA (N1–N11) subtypes of influenza A viruses, that potentially form 144 HA and NA combinations [1,2,3,4,5,6,7]. Aquatic birds including ducks, geese, and swans, are considered to be the natural reservoir of these subtypes.

Each year influenza viruses, both influenza A and influenza B are responsible for seasonal epidemics accounting for over 200,000 hospitalizations and 30,000–50,000 deaths [8,9,10,11,12]. As per World Health Organization (WHO) estimates, influenza viruses infect between 5%–15% of the global population, annually resulting in 250,000 to 500,000 deaths, making it the leading cause of mortality after acquired immune deficiency syndrome (AIDS). In addition to annual seasonal epidemics H1N1 and H3N2 viruses have also resulted in four major influenza pandemics: The “Spanish flu” in 1918, the “Asian flu” in 1958, the “Hong Kong flu” in 1968, and the more recent 2009 H1N1 pandemic. Since 1997, human infections with a novel H5N1 subtype of highly pathogenic avian influenza (HPAI) have been reported [13,14]. The first cases of human infection with H5N1 influenza were reported in 1997, when HPAI outbreaks in poultry farms and markets in Hong Kong resulted in eighteen cases and six deaths. Since then, this virus has spread to many countries in Asia, Africa, and Europe, resulting in over 424 human cases with a mortality rate greater than 60%. In addition, H9N2 and H7N7 avian influenza subtypes have also been reported to cause human infections [15,16]. The most recent strain infecting humans was H7N9 in China, detected in 2011 [17].

A number of diagnostic techniques, including virus isolation, nucleic acid amplification test (NAAT), immunochromatography-based rapid diagnostic test (RDT), *etc.*, have been used for detection of influenza viruses in humans. Here, we review various approaches currently available or under development for diagnosis of influenza infections in humans.

## 2. Virus Isolation Using Cell Culture Approaches

### 2.1. Viral Culture

Recovery of influenza viruses in clinical samples by propagation in mammalian cells or embryonated eggs is the most traditional method for influenza diagnosis. Introduced in the 1940s, the viral culture approach is considered one of the gold standards for the diagnosis of viral infections. This approach involves inoculation of permissive cell lines or embryonated eggs with infectious samples, propagation for 7–10 days to monitor development of cytopathic effect, and final confirmation of influenza virus infection by specific antibody staining, hemadsorption using erythrocytes, or immunofluorescence microscopy. Influenza virus isolation using this approach is usually performed on established cell lines, such as Madin Darby canine kidney (MDCK), A549, mink lung epithelial cell line (Mv1Lu), rhesus monkey kidney (LLC MK2), and buffalo green monkey kidney (BGMK), or primary cell lines, such as rhesus monkey kidney (RhMK) or African green monkey kidney (AGMK). In some labs, viral culture is used in parallel with NAAT assays.

### 2.2. Shell Viral Culture (SVC)

SVC is another viral culture approach that has been in use since the early 1990s for clinical diagnosis of influenza virus infections. This approach involves propagation of viruses in mammalian cells grown in small 1-dram vials or shell vials, followed by staining with influenza virus-specific fluorescent monoclonal antibodies. The SVC approach is relatively straightforward and more sensitive compared to traditional viral isolation method with viral detection possible in 24–48 h. A modified SVC method using R-mix cells (a mixture of mink lung cells and human adenocarcinoma cells) has demonstrated higher sensitivity compared to SVC approach with a turnaround time of approximately 1.4 days.

## 3. Direct Fluorescent Antibody Test (DFA)

The DFA test, also known as the immunofluorescent antibody test (IFA), is an antigen-based test routinely employed for diagnosis of influenza virus infections. In use since the early 1960s, this approach involves direct staining of respiratory epithelial cells derived from nasopharyngeal swabs or nasopharyngeal aspirates with fluorescently labeled influenza virus-specific antibodies followed by examination under a fluorescent microscope. Due to the simplicity of the assay procedure and the short turnaround time, DFA tests have popularly been used for influenza diagnosis. There are currently two Food and Drug Administration (FDA) approved DFA tests available in the market. They include the D3 FastPoint L-DFA test licensed to Diagnostic Hybrids (Athens, OH, USA), and Bartels Viral Respiratory Screening and Identification Kit licensed to Intracel Corporation (Issaquah, WA, USA). These tests are mainly intended for the detection and differentiation of influenza A and B viruses. However, they are not useful for subtyping influenza A viruses. For seasonal influenza viruses, DFA tests have demonstrated sensitivities ranging from 60%–80% compared with the traditional viral isolation procedures. During the 2009 H1N1 pandemic, DFA tests had demonstrated variable sensitivities, ranging between 38% and 93%, compared to reverse transcription polymerase chain reaction (RT-PCR) based approaches. A study comparing the performance of multiple tests, including DFA, virus isolation, and Quidel QuickVue Point of Care (POC) test with RT-PCR, for 2009 H1N1 pandemic virus (pH1N1) detection in 526 respiratory specimens reported assay sensitivities of 38.7% for DFA, 45.7% for virus isolation, and 18.2% for the QuickVue POC test [18]. Another study comparing the performances of DFA, two rapid influenza diagnostic tests (RIDTs); BinaxNOW Influenza A&B test (BinaxNOW), the 3M Rapid Detection Flu A + B test (3MA + B), R-Mix culture, and the Luminex xTAG Respiratory Virus Panel (RVP) for the detection of seasonal influenza, 2009 pandemic H1N1, and other respiratory viruses, reported assay sensitivities of 46.7% for DFA; 17.8% for BinaxNOW; 94.5% for R-Mix culture and 97.8% for RVP [19]. Another study, evaluating the performance of four tests (DFA, chromatographic influenza A and B virus immunoassay and shell vial culture) compared to the viral isolation approach reported assay sensitivities of 80% for DFA, 70.3% for rapid tests and 98.6% for shell vial culture [20]. A modified cytospin-enhanced DFA has been used by a few groups to enhance the sensitivity of DFA for influenza diagnosis. This approach uses a cytospin protocol to reduce inadequate smears and improve cell morphology to increase assay sensitivity and specificity. For detection of influenza A virus in clinical samples, the cytospin DFA test showed an assay sensitivity of 92.5% (49/53) compared with the cell culture assay. DFA tests are now being replaced around the world by molecular approaches that are more sensitive and less laborious for clinical diagnostic laboratory use. 

## 4. Serological Assays

Serological assays most commonly used to detect influenza virus-specific antibody responses include hemagglutination inhibition assay (HAI), microneutralization or virus neutralization assay (VN), single radial hemolysis (SRH), complement fixation assay, enzyme linked immunoabsorbant assay (ELISA) and Western blotting.

### 4.1. Hemagglutination Inhibition Assay

The HAI assay is the most commonly used method to determine the presence of influenza virus HA-specific antibodies in serum following natural infection and vaccination in humans and other animal models. This assay is based on the ability of HA-specific antibodies to prevent attachment of the influenza virus (approximately four hemaggulutinating units) to erythrocytes obtained from either chicken, turkey, human, horse, or guinea pigs. The highest serum dilution that prevents complete hemagglutination is called the HAI titer of the serum. Although this assay is simple and inexpensive, its sensitivity for identification of avian influenza A viruses, especially from the H5N1 subtype, has been shown to be poor, limiting its usability for virus diagnosis [21].

### 4.2. Virus Neutralization Assay

The VN assay is another technique used to measure induction of virus-specific antibodies following natural infection or vaccination, and is routinely used for the detection of antibody titers of either seasonal or avian influenza A virus strains. This approach is based on the ability of virus-specific antibodies to neutralize virus, thereby preventing viral infection of cells. The reciprocal of the highest serum dilution at which virus infection is completely blocked is considered the virus neutralization titer. Although the VN assay is more sensitive compared to the HAI assay, its application for routine diagnostic application is restricted due to the need of use of infectious viruses in certified BSL2+ and BSL3 laboratories [21].

### 4.3. Single Radial Hemolysis

SRH is a technique commonly used to determine the induction of antibodies following natural infection or vaccination. It is used to measure complement-mediated hemolysis induced by antigen-antibody complexes. SRH is considered more sensitive than the HAI assay and does not require pretreatment of the serum to inactivate non-specific inhibitors.

### 4.4. Complement Fixation

This is an immunodiffusion-based approach used to measure antibody responses to influenza virus internal proteins NP and M, following vaccination or infection [22]. Due to lower sensitivity, complement fixation has been replaced by HAI, VN, and EIA assays.

### 4.5. Enzyme Linked Immunoabsorant Assay

ELISA-based tests are available in either a solid microtiter plate format or a paper strip format. Rapid tests are based on the paper ELISA format and are discussed in detail in the section on rapid tests. ELISA-based tests were first introduced for disease diagnoses in the early 1990s and have consistently demonstrated high sensitivity and specificity. Several FDA-approved ELISA-based tests are available for the diagnosis of several bacterial and viral infections. Although ELISA-based tests have been in use for some time now, one major limitation has been the lower sensitivity compared to nucleic acid-based tests (NATs). Our laboratory has been evaluating approaches to enhance the sensitivity of antigen-based assays using europium and gold nanoparticles.

We developed a novel europium nanoparticle-based immunoassay (ENIA) for rapid detection of influenza viruses using monoclonal antibodies directed against the nucleoprotein from influenza A and influenza B viruses [23]. ENIA efficiently detected twenty-nine different strains of the influenza A subtypes H1, H2, H3, H5, H7, and H9, and ten different strains of influenza B. Furthermore, ENIA showed up to 16-fold higher sensitivity than a commercially available photometric ELISA for the various strains of influenza A and B viruses tested. While testing nasopharyngeal clinical samples from the 2012–2013 flu season ENIA demonstrated a sensitivity of 90.7% (147/162) for influenza A viruses and 81.80 % (9/11) for influenza B viruses with 100% specificity.

## 5. Rapid Influenza Diagnostic Tests (RIDTs)

RIDTs are antigen-based tests developed for rapid diagnosis of influenza virus infections in POC settings. These tests use monoclonal antibodies that target the viral nucleoprotein and employ either enzyme immunoassay or immunochromatographic (lateral flow) techniques. Available in dipstick, cassette, or card formats, RIDTs can be completed in less than 30 min, with the results observed visually based on a color change or other optical signals. Due to simplicity in their use and the speed of obtaining assay results, RIDTs are commonly used for the diagnosis of influenza infections.

Several FDA-approved RIDTs are currently available on the market. Most of these tests can either detect or distinguish influenza A and B viruses, detect only influenza A viruses, or both influenza A and B viruses (but cannot discriminate influenza A and B). However, none of the RIDTs can distinguish between the different influenza A subtypes. Performance of RIDTs is dependent on the prevalence of circulating influenza viruses in the population [24,25]. During peak influenza activity, positive predictive values are high and false positives are, therefore, likely to be observed. However, during low influenza prevalence, negative predictive values are high, with low positive predictive values. RIDTs have generally demonstrated high specificities (95%–99%) for the detection of seasonal influenza virus infections.

For diagnosis of seasonal influenza infections, RIDTs have demonstrated variable assay performance with sensitivities ranging between 10%–70%, with up to 90% specificity compared to standard RT-PCR-based assays. Performance of RIDTs have been shown to be better in children compared with adults (approximately 13% higher), potentially due to higher viral loads and longer viral shedding in children compared with adults [24]. A meta-analysis of 159 studies involving 26 commercial RIDTs showed a sensitivity of 62.3% compared to RT-PCR approaches for diagnosis of influenza infections (both seasonal and pH1N1 virus infections) [26]. In this study, the performance of RIDTs for the detection of influenza A viruses was higher compared to influenza B viruses (percentage sensitivities were influenza A: 64.6% and influenza B: 52.2%).

During the 2009 H1N1 pandemic, RIDTs demonstrated sensitivities ranging between 10%–70% compared with RT-PCR-based assays [27,28,29,30]. Using the BinaxNOW rapid antigen-based assay (Inverness Medical, Cologne, Germany), Drexler *et al*. reported an assay sensitivity of 11.1% while testing 144 PCR-positive clinical samples from Bonn, Germany [31]. Early during the pandemic, a large study from New York reported 9.6% and 40% sensitivities using RIDTs BinaxNOW Influenza A&B test (BinaxNOW), 3M Rapid Detection Flu A+B test (3MA+B) compared to R-Mix culture [19,32]. The low sensitivity of RIDTs during the pandemic could be attributed to poor sample quality and inexperience of the lab workers. A Centers for Disease Control and Prevention (CDC) study evaluating the performance of three different RIDTs (BinaxNOW Influenza A&B, Directigen EZ Flu A+B, and QuickVue Influenza A+B) for the detection of the pH1N1 virus had reported assay sensitivities of 40% for BinaxNOW Influenza A&B, 49% for Directigen EZ Flu A+B, and 69% for QuickVue Influenza A+B compared to a RT-PCR-based assay [33]. Due to a high rate of false negatives, the CDC advised physicians not to discontinue antiviral therapy despite negative RIDT results. In another study, the QuickVue Influenza RIDT assay (Quidel, San Diego, CA, USA) showed an assay sensitivity of 51% in comparison with a PCR assay [34]. Another study compared a RIDT QuickVue Influenza test with the RT-PCR-based assay and reported an assay sensitivity of 66% with 84% specificity [30]. The positive and negative predictive values in this study were 84% and 64%, respectively. A study comparing RIDT and cell culture approaches with a multiplex respiratory viral assay (Luminex xTAG), reported a combined assay sensitivity of 17.8% for the Binax NOW Influenza A+B (Inverness, Scarborough, ME) and the 3M Rapid Detection Flu A+B (3M Medical Diagnostics, St. Paul, MN, USA) [28]. While testing nasopharyngeal aspirates from 970 young children, one study reported 84.1% sensitivity using a RIDT, QuickVue Influenza A+B ICT test (Quidel Corp., San Diego, CA, USA) compared with a viral isolation method [35].

For detection of avian influenza A viruses, RIDTs have demonstrated lower sensitivity compared RT-PCR-based approaches. The FDA recently approved AV Avantage A/H5N1 Flu RIDT developed by Arbor Vita Corporation, (Sunnyvale, CA, USA) for H5N1 detection. This test uses a combination of monoclonal antibodies and PDZ (Post synaptic density protein (PSD95), Drosophila disc large tumor suppressor (Dlg1), Zonula occludens-1 protein (zo-1)) domain containing recombinant proteins to detect NS1 protein from throat swabs and can be completed in 45 min Although RIDT have demonstrated variable sensitivity, they still remain the test of choice in most clinical virology laboratories around the world due to the speed in obtaining results, simplicity in assay procedure, and cost.

## 6. Lab-on-a-Chip/Microchip Devices

A more versatile and powerful technology, lab-on-a-chip/microchip (LoC), provides a new route to develop a new generation of POC influenza tests. LoC technology originates from microelectromechanical system (MEMS) technology, with a focus on chemical and biological applications. It has many fascinating advantages, such as high reaction efficiency, low reagent/energy consumption, low waste generation, and a small footprint. LoC technology has been utilized to develop assays targeting multiple pathogens. With respect to influenza tests, Soh’s group reported a disposable microfluidic chip for sample-to-answer genetic analysis of H1N1 virus [36]. Bhattacharyya and Klapperich fabricated a plastic microfluidic solid phase extraction device to isolate viral RNA from mammalian cells infected with the influenza A (H1N1) virus [37]. In a recent report, Klapperich’s group described a disposable microchip integrated with solid-phase extraction and RT-PCR modalities capable of extracting and amplifying influenza A RNA directly from clinical specimens in less than three hours [38]. A continuous-flow microfluidic RT-PCR chip and disposable electrical printed (DEP) chips have been employed for rapid amplification and sensing of a swine-origin influenza virus. Using the RT-PCR chip method, the assay could be completed in 15 min and signals were detected with the DEP chip [39]. A miniaturized all-in-one, real-time RT-PCR instrument was developed, allowing automated sample preparation and diagnosis within 2.5 h. Typing and sub-typing of seasonal influenza A H1N1 was demonstrated using this system [40]. Another portable, user-friendly microchip NAT system was reported for POC diagnosis of influenza, with sensitivity close to that of a benchtop RT-PCR instrument. A microchip electrophoretic immunoassay coupled with laser-induced fluorescence detector was developed to detect swine influenza virus [41]. This system allows rapid and simultaneous concentration of viral particles and further separation of the virus-antibody complexes from the unbound antibody [42]. These influenza assays can be implemented using LoC technology and the microchip influenza assays exhibit impressive results. However, more efforts in further system improvements and assay validation are necessary to adapt the new LoC tests to real POC settings.

## 7. Nucleic Acid-Based Tests (NATs)

The development of the polymerase chain reaction (PCR) technique by Kary Mullis in 1983 revolutionized the field of infectious disease diagnosis. NAT (also known as NAAT) assays are based on PCR and detect virus-specific DNA or RNA sequences/ genetic material rather than viral antigens or antibodies. These tests are far more sensitive compared with the antigen-based serological tests, and can detect viruses much earlier in clinical samples. A variety of different NATs are currently available and used for diagnosis of influenza viral infections in humans. These include reverse transcriptase-PCR (RT-PCR), ligase chain reaction, sequencing-based tests (including pyrosequencing, next generation sequencing (NGS)), DNA microarray-based tests, nucleic acid sequencing-based amplification (NASBA), loop-mediated isothermal amplification-based assay (LAMP), simple amplification-based assay (SAMBA), transcription-mediated amplification, strand displacement amplification, *etc.* Most of these tests take 2–4 h to complete, demonstrating higher sensitivity and specificity compared with antigen-based tests. Currently, there are 21 FDA licensed NATs available for influenza diagnosis.

### 7.1. Reverse Transcriptase Polymerase Chain Reaction (RT-PCR)

RT-PCR is the most traditional yet powerful NAT approach for identification of influenza viruses in most diagnostic labs around the world. Considered a gold standard assay for influenza diagnosis, RT-PCR involves three essential steps: (1) extraction of viral RNA from clinical specimens; (2) Reverse transcription of viral RNA to a single-stranded cDNA using the enzyme reverse transcriptase; and (3) amplification of the PCR product is coupled to fluorescent detection of labeled PCR products.

### 7.2. Loop-Mediated Isothermal Amplification-Based Assay (LAMP)

LAMP is a DNA loop-mediated isothermal nucleic acid amplification approach that has been evaluated for detection of several viruses including severe acute respiratory syndrome (SARS) corona virus, rhinovirus, adenovirus, new castle disease virus, monkey pox virus, human immunodeficiency virus, and influenza virus. Initially developed by Notomo *et al*., this approach uses a novel DNA polymerase (or reverse transcriptase for RNA samples) with high strand displacement activity and two sets of primers specially designed to recognize six distinct regions on the viral cDNA. Specific amplification of the target gene is determined by either photometrically detecting the magnesium pyrophosphate by-product released in the solution at the end of the reaction or by observing the color change following addition of SYBR green. LAMP-based approaches have been successfully used for the detection of influenza viruses from clinical samples with sensitivity comparable to RT-PCR based assays. Using the LAMP-approach, Poon *et al*. reported 100% assay sensitivity for detection of seasonal influenza A viruses from subtypes H1N1 and H3N2 from clinical samples [43]. The analytical sensitivity of the assay was 10 copies per reaction. Furthermore, during the 2009 H1N1 pandemic, a real-time reverse transcription LAMP-based assay (RT-LAMP) demonstrated a sensitivity of 97.8% with 100% specificity for the pandemic virus compared to an RT-PCR-based assay [44]. LAMP-based assays have also been successfully evaluated for the detection of highly pathogenic avian influenza A viruses from subtypes H5N1, H7N7, and H7N9, with sensitivities comparable to, or even higher than, RT-PCR-based approaches [45,46,47]. Parida *et al.* developed a RT-LAMP assay targeting the gene coding for HA for clinical diagnosis of the pH1N1 virus [48]. The RT-LAMP assay performed better than a WHO-approved RT-PCR assay while testing 239 acute-phase throat swab samples from patients with influenza-like illness. It demonstrated up to 10-fold higher sensitivity compared to a WHO-approved RT-PCR method with an analytical sensitivity of 0.1 TCID_50_/mL (median tissue culture infective dose).

### 7.3. Simple Amplification-Based Assay (SAMBA)

SAMBA is a dipstick isothermal nucleic acid amplification approach, recently developed for the detection of HIV and influenza viruses. The approach involves a three-step procedure consisting of viral RNA extraction, target DNA amplification using an isothermal DNA polymerase and detection of the amplification product using a dipstick-based system. The SAMBA procedure takes approximately two hours to complete. Clinical performance of this approach has been evaluated for both seasonal and avian influenza viruses. While evaluating nasal/throat and nasopharyngeal swab specimens from 328 patients from the United Kingdom and Belgium, Wu *et al.* reported a sensitivity of 100% and 97.9%, respectively, for influenza A and B viruses compared to an RT-PCR approach [49]. The analytical sensitivity using this approach was 95 and 85 copies of viral genomes for influenza A and B viruses, respectively. In another study, the same group had reported an assay sensitivity of 95.3% with 99.4% specificity for the pH1N1 virus compared to a RT-PCR-based approach, based on testing RNA samples extracted from nasal/throat swab specimens from 262 patients [50].

### 7.4. Nucleic Acid Sequence-Based Amplification (NASBA)

NASBA is an isothermal PCR-independent amplification method that uses a combination of three enzymes: avian myeloblastosis virus reverse transcriptase, RNAse H, and T7 RNA polymerase in a single reaction. NASBA has been successfully evaluated for detection of both seasonal influenza A and highly pathogenic avian H5N1 and H7N9 avian influenza A viruses. Moore *et al.* evaluated NASBA for evaluation of H5N1 infection in 19 clinical samples obtained from confirmed cases of influenza A H5N1 infection in China. The assay demonstrated an analytical sensitivity of 0.01 TCID_50_ for A/VietNam/1194/2004 H5N1 virus, demonstrating an assay sensitivity of 100% [51]. In another study, Ge *et al.* used NASBA for rapid detection of the novel swine origin pH1N1 virus [52]. In that study, NASBA demonstrated an assay sensitivity of approximately 50 copies per reaction, which was comparable or higher than that observed with a commercial swine origin influenza A virus (S-OIV) (H1N1) real-time RT-PCR kit and CDC TaqMan assay. Recently, Wang *et al.* developed a modified NASBA procedure, referred to as a simple method for amplifying RNA targets, or SMART, for detection of seasonal H1N1 and H3N2 and pH1N1 viruses [53]. This isothermal amplification approach utilized single-stranded DNA (ssDNA) probes to serve as reporter molecules for capturing specific viral RNA (vRNA) sequences that are subsequently separated on a microfluidic chip under zero-flow conditions. The SMART assay demonstrated an analytical sensitivity of up to 10^5^ vRNA copies/mL with an assay sensitivity of 98.3% and specificity of 95.7% for detection of influenza A viruses.

### 7.5. Microarray-Based Approaches

Microarray-based approaches have proven to be useful tools for detection and subtyping of influenza viruses. For example, the FluChip microarray, a low-density DNA microarray, has been shown to detect H1N1, H3N2 and H5N1 strains in a few hours [54,55,56]. Similarly, a MChip microarray demonstrated 95% sensitivity and 92% specificity to identify influenza A virus. A semiconductor-based Influenza A microarray developed by CombiMatrix Corporation (Irvine, CA, USA) has been shown to detect all known subtypes of influenza A viruses in less than five hours [57,58]. A NanoChip 400 system (Nanogen Inc., San Diego, CA, USA) low-density microarray that employs one probe for the conserved gene coding for M and 97 probes for the cleavage site region of HA gene, was shown to be a useful diagnostic tool for the H5N1 virus [59]. However, all of these microarray-based assays require two or more enzymatic amplification steps of influenza viral RNA prior to hybridization. Moreover, detection requires labeling of multiple probes or incorporation of fluorescent dye- or biotin-conjugated nucleotides into double-stranded DNA (dsDNA) generated by RT-PCR. Furthermore, the sensitivities of most conventional microarray assays have been shown to rely on the efficiency of target amplification and hybridization of amplicons and probes. The multiple steps involved in these assays make them complex, expensive, labor intensive, susceptible to contamination, and also make them prone to false negative results due to the presence of gene mutations, PCR inhibitors, and RNA degradation. The design of multiple, virus-specific primer sets and assay optimization procedures pose diverse challenges.

Over the years, there has been growing interest in the use of nanoparticles coupled with silver staining for diagnostic applications due to the greater sensitivities compared with the fluorescent dyes that are commonly used in most microarray assays. This modified approach allows rapid detection of single nucleotide polymorphisms (SNPs) in human genomic DNA samples without the need for amplification of target template. In our laboratory, we developed a nanoparticle-based genomic microarray assay (nanomicroarray) that specifically identifies H5N1 viral nucleic acid and simultaneously provides subtype identification of influenza A virus in the absence of RT-PCR amplification procedures [60,61,62]. The nanomicroarray system has a high degree of hybridization efficiency and assay specificity. The method is simple and uses multiple oligonucleotides specially designed to target conserved regions from the entirely whole genome of genes coding for M, HA, and NA of influenza A and B viruses. In this approach, vRNA is allowed to bind to multiple capture, target, or intermediate oligonucleotides, and are hybridized to special gold nanoparticles. This multiplex oligonucleotide-based approach ensures the detection of newly-emerging strains that have undergone genetic assortment. The nanomicroarray assay can be used to detect influenza A and B viruses and also to differentiate different influenza A virus strains (H5N1 and pH1N1) from seasonal influenza. It can also simultaneously detect subtypes of influenza A viruses in a single assay using multiple samples per slide. The analytical sensitivity of the nanomicroarray is <100 fM using purified PCR fragment and 1 × 10^3^ TCID_50_ units for H5N1 vRNA. The nanomicroarray images can be scanned for further analysis, and conclusions on virus identity can be drawn from results seen as a specific intensity pattern using the naked eye. In combination with a next generation sequencing assay, this assay is a minimally-manipulated procedure that greatly reduces the number of amplifications, and omits fragment separation and purification. It is therefore suitable for screening and identification of a broad range of subtypes of influenza virus in a time- and labor-efficient manner to achieve final laboratory confirmation. The new influenza detection algorithm provides a new way to refine differential diagnoses for identification of infection risks of unknown influenza strains by selecting a single test, or a small set of tests to determine the HPAI strain in clinical samples. This method provides a new tool for accurate diagnoses and rapid identification during seasonal epidemics or pandemics [60].

## 8. Nucleic Acid Sequencing Approaches

### 8.1. Sanger Sequencing

Sanger sequencing, also referred to as the dideoxy procedure, is a chain termination method of DNA sequencing. Developed by Fredrick Sanger and colleagues in the late 1970s, this approach involves the use of DNA polymerase, a pair of DNA primers, unlabeled deoxynucleotide triphosphates (dNTPs), and chain-terminating dideoxynucleotides (di-ddNTPs) with each base labeled with a unique fluorophore. Here, selective incorporation of di-ddNTPs into a newly synthesized strand by DNA polymerase inhibits addition of subsequent dNTPs preventing further elongation of the target DNA resulting in DNA fragments of various sizes containing fluorescently labeled ddNTP at the end of the strand. The DNA sequence is then determined based on the size of DNA fragments from the sequencing reaction. This sequencing approach can generate 1000 bp DNA sequence reads per reaction. Sanger sequencing has been widely used for whole genome sequencing of influenza viruses. Moreover, Sanger sequencing has also been used for the detection of antiviral resistance among circulating influenza viruses.

### 8.2. Next-Generation Sequencing (NGS)

NGS is a rapidly expanding technology and the techniques may potentially generate enormous impacts on life science, medicine, and related fields. NGS offers significant improvements in sequencing speed and throughput when compared with Sanger sequencing. Its automated streamline workflow greatly simplifies sample preparation. Since NGS directly analyzes nucleic acid fragments extracted from samples, it obviates the complex and time-consuming vector cloning imperative to Sanger sequencing. An added merit of NGS is that of the sequencing cost. The expense for sequencing a human-sized genome using Sanger sequencing decreased from 100 million US dollars in 2001 to about 5000 dollars in 2014 [63], and is expected to decrease further in the near future. Many applications based on NGS techniques have emerged in recent years, and we focus mainly on use in diagnosis of influenza virus in this review. We encourage readers to explore other dedicated reviews, research articles, or company websites for more detailed information on NGS platforms.

NGS technology is comprised of several manufacturer-specific platforms that use different sequencing strategies, reagents, and bioinformatics software. Roche 454 Life Sciences (Branford, CT, USA) developed NGS platforms based on pyrosequencing, a high-throughput sequencing-by-synthesis bioluminescence method that monitors real-time release of pyrophosphate following incorporation of dNTPs into a growing strand of nascent DNA. This sequencing platform has been evaluated as a diagnostic tool in influenza research by several groups, primarily for identification of molecular markers of drug resistance among circulating influenza A viruses. Bright and colleagues were the first to use this platform to study the incidence of adamantine resistance among seasonal influenza A viruses from subtypes H1N1, H1N2, and H3N2, isolated worldwide from 1994–2005 [64]. During a decade of surveillance, a significant increase in drug resistance, from 0.4% in 1994–1995 to 12.3% was observed in 2003–2004. This study highlighted the clinical importance of rapid surveillance for antiviral resistance among circulating seasonal influenza virus strains. Since then, several groups have successfully used the pyrosequencing platform to identify mutations in genes coding for M2 and NA responsible for resistance to adamantanes, amantadine and rimantadine and oseltamavir, respectively, among seasonal and highly pathogenic H5N1 avian influenza viruses. Pyrosequencing has also been used by a few groups to identify SNPs in the gene coding for hemagglutinin. Levine *et al*. used a customized pyrosequencing protocol for the detection of pH1N1 virus variants at amino acid position 222 of hemagglutinin, known to be associated with enhanced virulence in humans [65]. More recently, Lee *et al.* used pyrosequencing to identify G186D and R292K mutations in genes coding for HA and NA in an immunocompromised child infected with influenza A/H3N2 virus being treated with oseltamivir [66]. Roche 454 NGS instruments are no longer in use, following the shutdown of 454 Life Sciences in October 2016.

Illumina’s NGS platforms use different sequencing-by-synthesis approaches based on amplification of nucleic acid fragments on solid substrate or bridge amplification. Using an Illumina MiSeq sequencer (San Diego, CA, USA), Rutvisuttinunt *et al.* acquired complete genome sequence information from influenza virus isolates, and identified seasonal influenza A H3N2, p H1N1 and influenza B viruses simultaneously [67]. Ren *et al*. applied an Illumina/Solexa GAII sequencer to sequence the full genome of the recently emerging influenza A H7N9 strain without virus culture [68]. Kuroda *et al.* sequenced the RNA extracted from an autopsy lung of an influenza-pH1N1 infected patient using an Illumina GAII sequencer. They performed *de novo* assembly and identified possible co-infections caused by bacterial such as *Streptococcus* *pneumonia* [69]*.* Greninger *et al*. employed both ViroChip microarray and Illumina NGS techniques in the diagnosis of multiple patient samples (metagenomic strategy) [70]. They showed that use of ViroChip microarray allowed the correct detection of influenza pH1N1 without *a priori* information, while the NGS deep sequencing technique provided in-depth knowledge into the upper respiratory microbiota and host gene expression in response to virus infection. Whitehead *et al*. utilized Illumina NGS platform to obtain comprehensive sequence-function map through deep sequencing, and optimized influenza-binding proteins based on the NGS data [71]. A combination of this approach and pre-existing computational drug design may open up a new avenue to more efficiently develop novel influenza inhibitors.

There are a few alternative NGS platforms, besides the Roche 454 and Illumina MiSeq platforms, that may be suitable for influenza diagnosis, including Life Technologies/Applied Biosystem’s Ion personal genome machine (PGM), Ion Proton and SOLiD NGS platforms, and Pacific Biosciences’ PACBIO RS/RSII single-molecule, real-time sequencing platform. Each platform has its own advantages and disadvantages, as the underlying proprietary sequencing techniques are quite different. It is therefore crucial for researchers to select the most appropriate NGS platforms to meet their specific needs, based on factors including throughput, read length, error rate, cost, *etc.* A recent comparison study reported by Quail *et al*. may provide some insightful NGS platform selection criteria [72]. Three platforms, Ion PGM, PACBIO RS, and Illumina’s MiSeq, were employed to sequence four microbial genomes with mean GC content ranging from 19.3% to 67.7%. Coverage distribution, bias, GC distribution, variant detection, and accuracy were used as figures of merit in the comparison. The authors concluded that all three platforms provide near 100% coverage on GC-rich, neutral and moderately AT-rich genomes. However, systematical bias was observed on sequencing the extremely AT-rich genome of *Plasmodium falciparum* on the PGM. Context specific errors were identified in both Ion PGM and MiSeq data, but not in that from PACBIO RS. Additionally, PACBIO RS has limited yield and high cost per base, prohibiting its use in large-scale sequencing. The Ion PGM and MiSeq are equivalent in utility and ease of workflow.

More recently, our lab has demonstrated a “one-test-fits-all” approach to simultaneously identify any unknown influenza infections and co-infections in a single-tube, using a universal RT-PCR-NGS diagnostic platform to characterize influenza whole-genome for putative antiviral resistance and high virulence markers that may confer high virulence in human hosts. By testing 176 clinical specimens, we demonstrated that this platform can achieve highly sensitive detection equivalent to the clinical real-time RT-PCR (rRT-PCR) test at low virus concentrations, and allows multiplex identification and simultaneous discrimination of functional significance by providing the whole spectrum of the influenza genome. A typical genetic matrix composition from a known and a novel emerging influenza infection can be determined [73]. Development ofNGS-based detection will help to uncover new levels of innovation and efficiency, which will facilitate future use of an NGS diagnostics platform for public health surveillance.

## 9. Conclusions

Antigenic drift resulting from accumulation of point mutations in the viral genome generates novel variants that escape immunity to previous influenza strains causing annual seasonal epidemics. Genetic assortment between human, swine, or avian influenza strains has been shown to result in emergence of influenza viruses with novel HA and/or NA genes, against which the majority of human population lacks immunity. Diagnostic techniques and approaches that can rapidly and accurately detect newly emerging viral variants are required for quick initiation of antiviral therapy and prophylaxis to effectively control infection during seasonal and pandemic outbreaks. NATs have demonstrated high specificity and sensitivity for detection of influenza viruses. However, they are less practical in resource-limited regions due to their high cost, instrumentation complexity, requirement for well-maintained environment and highly trained professionals. A large number of low-cost, portable, point-of-care RIDTs based on multiple mechanisms have been developed to meet the demands for rapidly diagnosing epidemic or pandemic influenza in remote settings. Unfortunately, RIDTs have demonstrated variable sensitivity for diagnosis of both seasonal and pandemic influenza virus infections. Furthermore, most of the current FDA-licensed tests for influenza can detect and differentiate influenza A and B viruses, but have a limited capability to further subtype influenza A viruses. Hence, newer approaches that are cost-effective, less labor intensive, easy to perform, and have capacity to both detect and differentiate influenza viruses, while also subtyping influenza A viruses, are currently a global public health requirement. There has been a steady increase in resistance to currently used M2 and NA inhibitors among circulating seasonal and novel avian-origin H5N1 and H7N9 influenza A viruses, highlighting the need for molecular surveillance of putative virulence factors and antiviral resistance markers to improve public health by implementing appropriate diagnostic and treatment strategies during seasonal outbreaks and epidemics.

## Abbreviations

The following abbreviations are used in this manuscript: M: matrix protein; NA: neuraminidase; NP: nucleoprotein; HA: hemagglutinin; NS: non-structural protein; vRNA: viral RNA; HPAI: highly pathogenic avian influenza; pH1N1: 2009 H1N1 pandemic virus; RVP: respiratory virus panel; SRH: single radial hemolysis; ELISA: enzyme linked immunoabsorbant assay; NAATS: nucleic acid amplification tests; RDTs: immunochromatography-based rapid diagnostic tests; SVC: shell viral culture; DFA: direct fluorescent antibody test; IFA: immunofluorescent antibody test; HAI: hemagglutination inhibition assay; VN: virus neutralization assay; ENIA: europium nanoparticle-based immunoassay; POC: Point of Care; LoC: lab-on-a-chip/microchip; PCR: polymerase chain reaction; NATs: nucleic acid-based tests; NASBA: nucleic acid sequencing-based amplification; RT-PCR: reverse transcriptase polymerase chain reaction; LAMP: loop-mediated isothermal amplification-based assay; SAMBA: simple amplification-based assay; CDC: Centers for Disease Control and Prevention; SNPs: single nucleotide polymorphisms; NGS: next-generation sequencing; RIDTs: rapid influenza diagnostic tests; SARS: severe acute respiratory syndrome.
